# Herbivore-Induced Rice Volatiles Attract and Affect the Predation Ability of the Wolf Spiders, *Pirata subpiraticus* and *Pardosa pseudoannulata*

**DOI:** 10.3390/insects13010090

**Published:** 2022-01-13

**Authors:** Jing Liu, Liangyu Sun, Di Fu, Jiayun Zhu, Min Liu, Feng Xiao, Rong Xiao

**Affiliations:** Guizhou Provincial Key Laboratory for Agricultural Pest Management of the Mountainous Region, Institute of Entomology, Guizhou University, Guiyang 550025, China; lj1071836108@163.com (J.L.); liangysun@126.com (L.S.); fudi9696@163.com (D.F.); zjy873421380@163.com (J.Z.); liumin8796@163.com (M.L.); xiaofeng1217@163.com (F.X.)

**Keywords:** rice field spiders, herbivore-induced rice volatiles, selection behavior, predation latency, daily predation capacity

## Abstract

**Simple Summary:**

The spiders, *Pirata subpiraticus* Bösenberg *et* Strand (Araneae: Lycosidae) and *Pardosa pseudoannulata* Bösenberg *et* Strand (Araneae: Lycosidae) are important natural enemies of many rice pests. Herbivore-induced plant volatiles can attract natural enemies to pest locations and are becoming important in integrated pest management. This study assessed the effects of herbivore-induced rice volatiles on the selection behavior, predation ability and field attraction of two species of spiders. The selection frequency of spiders for methyl salicylate, linalool, and 2-heptanone were significantly greater than the blank group. Methyl salicylate can shorten the predatory latency of male *P. pseudoannulata* and can also trap more *P. pseudoannulata* in the field. Linalool may also shorten the predatory latency of male *P. subpiraticus* and increase the daily predation capacity of female *P. pseudoannulata*. In summary, herbivore-induced rice volatiles attract *P. pseudoannulata* and *P. subpiraticus*, and potentially increase their pest control capability. These results provide support for the practical use of herbivore-induced rice volatiles to attract and retain spiders in rice fields.

**Abstract:**

Spiders are important natural enemies of rice pests. Studying the effects of herbivore-induced rice volatiles on spider attraction and predation ability may lead to safer methods for pest prevention and control. In this study, four-arm olfactometer, predation ability experiment, and field trapping experiment were used to evaluate the effects of herbivore-induced rice volatiles on *Pirata subpiraticus* Bösenberg *et* Strand (Araneae: Lycosidae) and *Pardosa pseudoannulata* Bösenberg *et* Strand (Araneae: Lycosidae). The 0.5 μg/μL linalool concentration was attractive, and also shortened the predation latency in male *P. subpiraticus* and female *P. pseudoannulata*. The 0.5 μg/μL linalool concentration increased the daily predation capacity of female *P. pseudoannulata*. Male *P. pseudoannulata* were attracted to 1.0 g/L methyl salicylate, which also shortened their predation latency. In field experiments, methyl salicylate and linalool were effective for trapping spiders. Herbivore-induced rice volatiles attract rice field spiders and affect their predatory ability. These results suggest that herbivore-induced rice volatiles can be used to attract spiders and provide improved control of rice pests.

## 1. Introduction

Rice is an important food crop and rice yield is always a high priority [1]. However, pests such as *Nilaparvata lugens* Stal (Homoptera: Delphacidae), *Sogatella furcifera* Horváth (Homoptera: Delphacidae), *Chilo suppressalis* Walker (Lepidoptera: Pyralidae), and *Cnaphalocrocis medinalis* Guenee (Lepidoptera: Pyralidae) can reduce rice production [2]. Pesticide application is the main approach for managing rice pests. Although chemical pesticides are effective, their widespread use has resulted in an increase in the occurrence of 3R (residue, resistance, and resurgence). This has made the biological management of rice pests more important [3,4,5]. Herbivore-induced plant volatiles (HIPVs) regulate the interaction of plants, herbivorous insects, and their natural enemies [6,7]. They can be used as attractants for the natural enemies of pests [6,8]. Herbivore-induced rice volatiles (HIRVs) such as (Z)-3-hexen-1-ol, methyl salicylate, 2-heptanone, linalool, and others are produced or increased when rice is damaged by pests [9,10,11,12,13]. HIRVs increase significantly, when rice is eaten by adult females of the brown planthopper *N. lugens* and the white-backed planthopper *S. furcifera* [14]. Some HIRVs are attractive to natural enemies of rice planthoppers, such as *Anagrus nilaparvatae* Pang *et* Wang (Hymenoptera: Mymaridae) [9,15,16], *Haplogonatopus japonicus* Esaki *et* Hashimoto (Hymenoptera: Dryinidae) [17] and *Cyrtorhinus lividipennis* Reuter (Hemiptera: Miridea) [18,19]. Some HIRVs are attractive to *Apanteles chilonis* Munakata (Hymenoptera: Braconidae), which is the natural enemy of *C. suppressalis* [20]. The HIRVs released by *Tibraca limbativentris* Stål (Heteroptera: Pentatomidae) and *Glyphepomis spinosa* Campos *et* Grazia (Heteroptera: Pentatomidae) feeding on rice are attractive to the natural enemy of rice pest, *Telenomus podisi* Ashmead (Hymenoptera: Platygastridae) [21].

Spiders are often referred to as the “Paddy Field Guardian”, and are natural enemies of rice pests in the rice fields of China [22,23]. Spiders are carnivorous, with a large food intake, strong predatory ability, high reproductive rate, and high adaptability [24]. Therefore, spiders play a significant role in the biological management of rice pests. In China, the dominant species of rice field spiders include *Tetragnatha maxillosa* Thoren (Araneae: Tetragnathidae), *Pardosa pseudoannulata* Bösenberg *et* Strand (Araneae: Lycosidae), *Pirata subpiraticus* Bösenberg *et* Strand (Araneae: Lycosidae), *Pirala piraticus* Clcrck (Araneae: Lycosidae), *Clubiona japonicola* Bösenberg *et* Strand (Araneae: Clubionidae), and *Oxyopes sertatus* L. Koch (Araneae: Oxyopidae) [25,26,27]. Netting spiders and wandering spiders are the two main types of dominant spiders. Netting spiders feed on a variety of flying insects while wandering spiders feed on insects in many locations [26]. Wandering wolf spiders are the majority spiders in rice fields. Wolf spiders rely on their sense of smell to discover and locate their prey [28,29]. Cao et al. identified the wolf spider *P. pseudoannulata* having two potential odorant-binding protein genes [30]. This information provides the basis for further research on the olfactory selection behavior of wolf spiders. Spider olfaction is critical in their predatory behavior [31], but it is not known if HIRVs are attractive to spiders. Therefore, we selected two paddy wolf spiders, *P. subpiraticus* and *P. pseudoannulata*, to study the selection behavior of spiders on HIRVs. *P. subpiraticus* often forages on rice, water, and land to prey on rice pests [32]. It has strong predation ability and starvation tolerance. It can consume 6–16 *S. furcifera* every 24 h [33], and survive up to 42.7 d without food under certain humidity conditions [34]. *P. pseudoannulata* is an important predator of rice pests. It is a hunting spider with a wide niche, good running and jumping ability, and strong predatory ability for rice pests [35].

In the study, two rice field spiders and four volatiles were selected to ascertain if HIRVs are attractive to spiders and to determine the optimum concentrations of the attractive volatiles. We used the optimum concentration to verify how HIRVs affect the predatory ability of spiders. HIRVs were also used as attractants to trap spiders in the field.

## 2. Materials and Methods

### 2.1. Spiders

*P. subpiraticus* and *P.* *pseudoannulata* were collected from a rice field in Yanlou Town, Huaxi District, Guiyang, China (106°6′24″ E, 26°3′19″ N). Spiders of various growth stages were collected and placed in individual plastic test tubes (12 cm × 3.5 cm diam). To maintain humidity, a water-soaked sponge was placed at the bottom of each test tube, and the top of the tube was sealed with a cotton ball. Spiders were raised in a clear artificial climate box at 25 °C ± 1 °C, 75% ± 5% relative humidity, and a 14:10 h (L:D) photoperiod. The spiders were fed *Musca domestica* adults twice a week (2–3 *M. domestica* adults each time) until they were adults.

### 2.2. Volatiles

The volatiles were purchased from Sigma-Aldrich (St. Louis, MO, USA). There were four volatile standard products, 99% methyl salicylate (MeSA), 98% cis-3-hexen-1-ol (CH), 99% 2-heptanone (HE), and 99% linalool (LI). The control was 99.9% liquid paraffin. The four volatiles were dissolved in liquid paraffin and diluted to test concentrations of 0.5 μg/μL, 1 μg/μL, and 1.5 μg/μL based on existing information on the spectrum of HIRVs [11,12].

### 2.3. Experimental Treatments

#### 2.3.1. Spider Selection Behavior Experiments in Response to HIRVs

**Figure 1:**
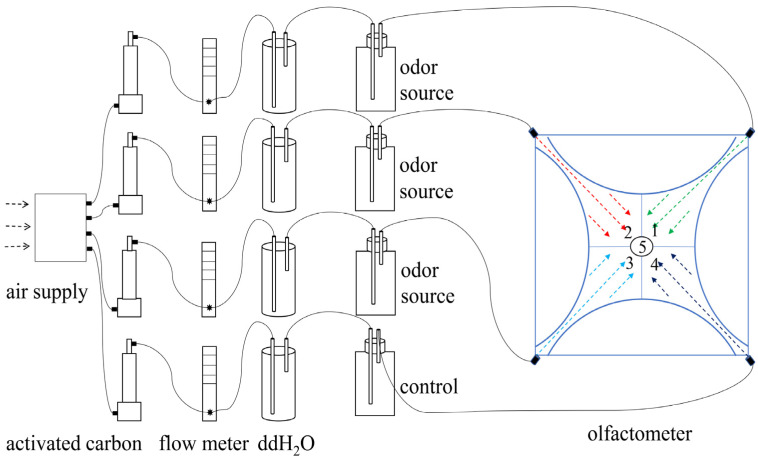
Experimental olfactometer for olfactory selection. The black wire connecting the instrument represents the rubber tube. In the olfactometer, the solid cross-line represents the boundary of the test area; 1, 2, 3, 4 are four test areas, and the dotted lines of different colors represent different test volatiles and flow directions; 5 is the introduction area of the spiders and the exit location of the volatiles.

A four-arm olfactometer was used following the procedure of Vet et al. [36,37] (Figure 1). The test cavity of the olfactometer was 15 cm in diameter and composed of transparent Plexiglas. Four odor areas and centers were drawn on the surface of the olfactometer’s test cavity. The purpose of the area boundaries was to determine spider location. Silicon tubes were used to link the odor source bottle (or control bottle), gas cleaning bottle, activated carbon filter bottle, flow meter, and atmosphere collector to the olfactometer. A 20 W fluorescent bulb was placed 30 cm above the olfactometer. A gas flow meter was used to regulate the airflow of the four arms at 400 mL/min. The indoor test temperature was 25 °C ± 3 °C.

The spiders were starved for 48 h before being tested (water was added without food). Liquid paraffin was used as the control and to dilute the volatile standards. Before starting the volatile test, we first observed the spider’s selection behavior of the four arms of the olfactometer for placed liquid paraffin. During the test, one of the four arms was defined as the control arm, while the other three were defined as the treatment arms. The filter paper (4 cm diam) was sprayed with 20 μL quantities of different concentrations of the same volatiles (20 μL liquid paraffin was used as a control) and placed in the olfactometer’s four odor source bottles. Second, we pumped the air for 5 min to fill the pipe with the odors then used a funnel to introduce the test spiders into the test chamber of the olfactometer where they were observed for 5 min. We recorded spider movements in each odor area and in the central area. If the spider entered the arm of a certain odor area and remained there for 2 min, the arm volatile was deemed the spider’s last choice. The spider was deemed unresponsive if it did not make a choice within 5 min after entering the test cavity. When the spider made a final choice in one of the arms, the remaining time (5 min minus the time spent making the final choice) was added to that arm. Each time, one spider was tested, and each volatile was tested on 30 female or male adults of each species. We changed the filter paper in the bottle after five tests were completed, cleaned the olfactometer with 100% ethanol, and dried it with a hair drier. We linked the olfactometer to each odor source bottle and the control bottle after each cleaning.

#### 2.3.2. Spider’s Predatory Ability Experiments

We used *P. subpiraticus* and *P. pseudoannulata* as experimental subjects to determine if HIRVs could affect the predatory ability of rice field spiders. The test spiders were starved for seven days before predation testing, since spiders can ingest and store surplus food. This was done to ensure that the spiders would be able to hunt during the experiments. During the experiment, we placed filter paper containing 3 μL of the volatile into the plastic tube with spiders for 30 min, then placed *Drosophila melanogaster* (prey, well-developed, and with similar body sizes) into the plastic tube with spiders. Preliminary experiments show that spiders of different sizes and sexes have different predatory abilities. Therefore, females of *P. pseudoannulata* placed 40 preys, and other spiders placed 30 preys. Twenty female or male adults of each spider species were tested for each HIRV, and the predatory latency (the period between the preys were placed and the spider successfully finished the attack), as well as daily predation was recorded.

#### 2.3.3. Field Trapping Experiments

Xixiu District, Anshun, Guizhou Province, China (106°9′19″ E, 26°9′38″ N) was selected as the site for the field experiment. The rice was in the filling stage. We selected volatiles such as methyl salicylate, linalool, and 2-heptanone, which were all attractive to spiders in experimental treatments. The attractant was formulated in an 8:1 proportion of volatiles to liquid paraffin. A 225 mL plastic cup served as the trap. The 2 mL attractant centrifuge tube was connected to the inside of the plastic cup with small holes drilled at the bottom and liquid paraffin was the control. We set traps between rice plants at the base of every 16 clusters of rice, 10 traps for each volatile. We recorded the species and the number of spiders in the traps after 7 days. In each HIRVs field experiment, there were three replications with a plot spacing of at least 10 m to minimize inter-plot interference.

### 2.4. Statistical Analysis

Experiment data were analyzed using IBM SPSS Statistics 21.0. The selection frequency of spiders between different concentrations of volatiles was tested using a goodness-of-fit χ^2^ test. The observed behavioral responses were compared to expected frequencies assuming a random distribution of spiders to volatiles (three concentration odor sources and one control). The stay (retention) time of spiders in different areas was compared using one-way analysis of variance (ANOVA). The attack latency and daily predation of spiders between different treatments were assessed using one-way ANOVA in the predation experiment. In the field experiment, one-way ANOVA was used to compare spider trapping rates between the different treatments.

## 3. Results

### 3.1. Spider Selection Behavior in Response to HIRVs

Liquid paraffin was used as the control and to dilute the volatile standards. The *P. pseudoannulata* and *P. subpiraticus* selection frequencies showed no significant difference between liquid paraffin in the four arms of the olfactometer (Table 1).

**Figure 2:**
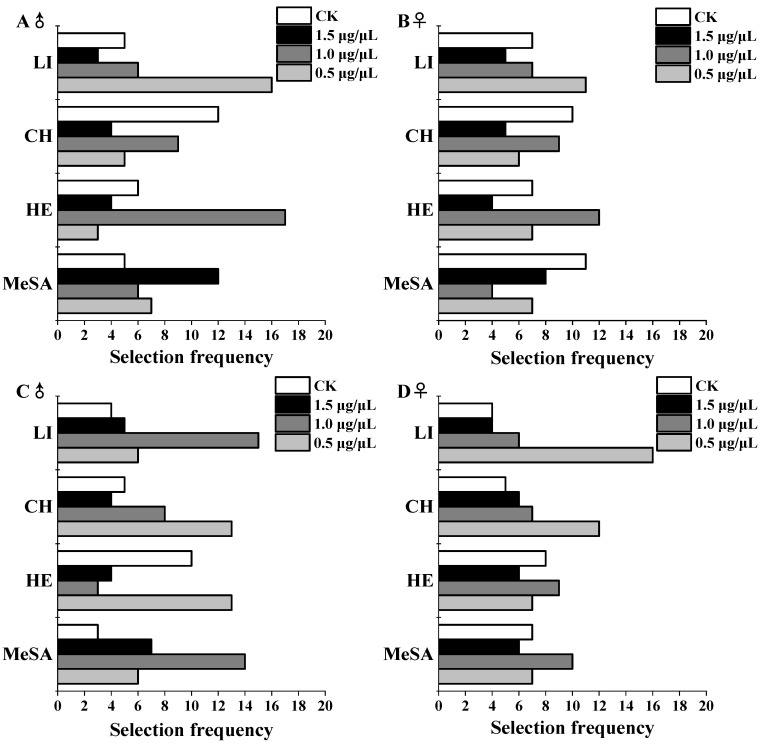
Selection frequency (N = 30), (**A**) male *P. subpiraticus*, (**B**) female *P. subpiraticus*, (**C**) male *P. pseudoannulata*, (**D**) female *P. pseudoannulata*; MeSA indicates methyl salicylate, HE indicates 2-heptanone, CH indicates cis-3-hexen-1-ol, LI indicates linalool.

Spider selection between three concentrations of the same volatile and a negative control was studied using a four-arm olfactometer. The results showed that selection frequency of male *P. subpiraticus* for 2-heptanone (χ^2^ = 16.667, *p =* 0.001) and linalool (χ^2^ = 13.467, *p* = 0.004) were significantly different (Table 1). The male *P. subpiraticus* showed a stronger preference for 0.5 μg/μL linalool and 1.0 μg/μL 2-heptanone than for other concentrations and controls (Figure 2). However, there was no significant difference in selection frequency for cis-3-hexen-1-ol (χ^2^ = 5.467, *p* = 0.141) and methyl salicylate (χ^2^ = 3.867, *p* = 0.276) (Table 1). The selection frequency of female *P. subpiraticus* for linalool (χ^2^ = 2.533, *p* = 0.469), 2-heptanone (χ^2^ = 4.400, *p* = 0.221), methyl salicylate (χ^2^ = 3.333, *p* = 0.343), and cis-3-hexen-1-ol (χ^2^ = 2.267, *p* = 0.519) were not significantly different (Table 1). The selection frequency for methyl salicylate (χ^2^ = 8.667, *p* = 0.034), 2-heptanone (χ^2^ = 9.200, *p* = 0.027), and linalool (χ^2^ = 10.267, *p* = 0.016) in male *P. pseudoannulata* were significantly different (Table 1). Male *P. pseudoannulata* selected 1.0 μg/μL linalool, 1.0 μg/μL methyl salicylate, and 0.5 μg/μL 2-heptanone more frequently than other concentrations and controls (Figure 2). The selection frequency for cis-3-hexen-1-ol (χ^2^ = 6.533, *p* = 0.088) in male *P. pseudoannulata* was not significant (Table 1). Female *P. pseudoannulata* selection frequency for linalool (χ^2^ = 13.200, *p* = 0.004) was significantly different (Table 1). The selection frequency of 0.5 μg/μL linalool by female *P.* *pseudoannulata* was higher than the other concentrations and controls (Figure 2). Female *P. pseudoannulata* selection frequency for methyl salicylate (χ^2^ = 1.200, *p* = 0.753), 2-heptanone (χ^2^ = 0.667, *p* = 0.881), and cis-3-hexen-1-ol (χ^2^ = 3.867, *p* = 0.276) were not significantly different (Table 1).

We studied the duration that spiders remained in different treatments to see if HIRVs affected their selection behavior. Male *P. pseudoannulata* remained significantly longer in the presence of 1.0 μg/μL methyl salicylate and 1.0 μg/μL linalool than in other concentrations of the same volatiles or the control treatment. Female *P. pseudoannulata* remained significantly longer in the presence of 0.5 μg/μL linalool than in the presence of other concentrations of the same volatile or the control treatment. Male *P. subpiraticus* remained longer with 1.0 μg/μL 2-heptanone and 0.5 μg/μL linalool than with other volatile concentrations or the control treatment (Table 2).

In summary, comprehensive selection frequency and stay time of *P. pseudoannulata* and *P. subpiraticus*, 1.0 μg/μL methyl salicylate and 1.0 μg/μL linalool were attractive to males of *P. pseudoannulata*; 0.5 μg/μL linalool was attractive to females of *P. pseudoannulata*; 1.0 μg/μL 2-heptanone and 0.5 μg/μL linalool were attractive to males of *P. subpiraticus.*

### 3.2. Spider’s Predatory Ability

#### 3.2.1. Daily Predation Capacity and Predatory Latency of *P. subpiraticus*

**Figure 3:**
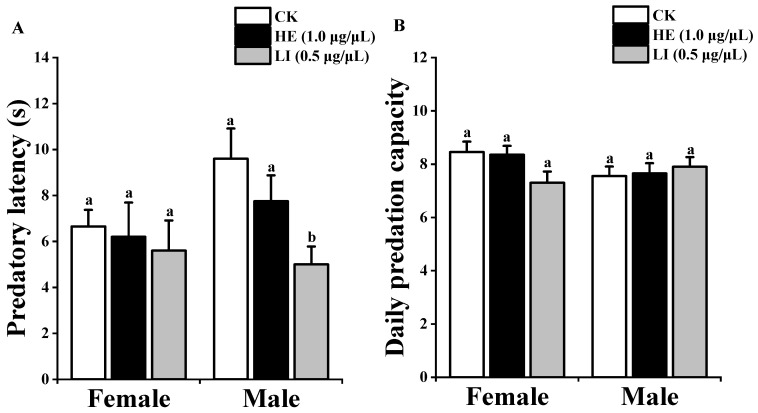
Predatory efficiency of *P. subpiraticus* on *Drosophila* *melanogaster*: (**A**) indicates the predatory latency of *P. subpiraticus*. (**B**) indicates the daily predation capacity of *P. subpiraticus.* CK indicates liquid paraffin, HE (1.0 μg/μL) indicates 2-heptanone (1.0 μg/μL), LI (0.5 μg/μL) indicates linalool (0.5 μg/μL). The data are expressed as mean ± SE. Vertical bars indicate SE. Columns of the same sex with different lowercase letters are significantly different (ANOVA, Tukey *p* < 0.05).

There was no difference in the predatory latency of female *P. subpiraticus* between treatments. The predatory latency of male *P. subpiraticus* was not significantly different between control and 2-heptanone (0.5 μg/μL), but linalool (0.5 μg/μL) treatment was significantly shorter than that of control and 2-heptanone (0.5 μg/μL). The daily predation capacity of female and male *P. subpiraticus* was not significantly different between the treatments (Figure 3).

#### 3.2.2. Daily Predation Capacity and Predatory Latency of *P. pseudoannulata*

**Figure 4:**
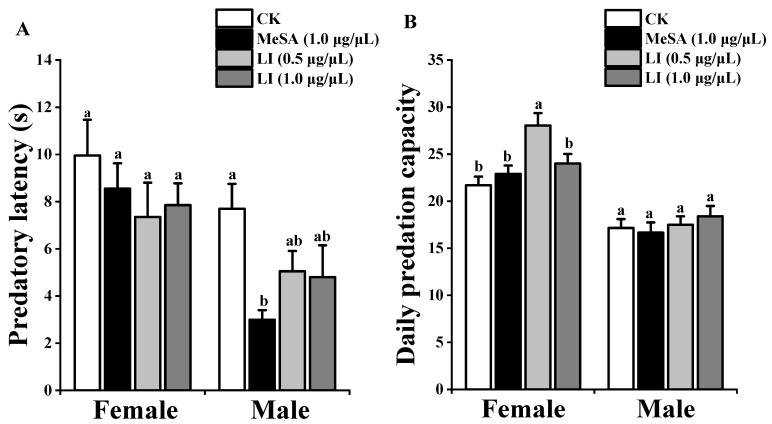
Predatory efficiency of *P. pseudoannulata* on *Drosophila* *melanogaster*: (**A**) = the predatory latency of *P. pseudoannulata*. (**B**) = the daily predation capacity of *P. pseudoannulata.* CK = liquid paraffin, MeSA (1.0 μg/μL) = methyl salicylate (1.0 μg/μL), LI (1.0 μg/μL) = linalool (1.0 μg/μL), LI (0.5 μg/μL) = linalool (0.5 μg/μL). The data are expressed as mean ± SE. Vertical bars = SE. Columns of the same sex starting with different lowercase letters are significantly different (ANOVA, Tukey *p* < 0.05).

The predatory latency of female *P. pseudoannulata* was not significantly different between the treatments. The predatory latency of male *P. pseudoannulata* was not significantly different between the control, linalool (1.0 μg/μL), and linalool (0.5 μg/μL). However, the 1.0 μg/μL methyl salicylate treatment predatory latency was significantly shorter than that of the control, linalool (1.0 μg/μL), and linalool (0.5 μg/μL). The daily predation of male *P. pseudoannulata* was not significantly different between the treatments. The daily predation capacity of female *P. pseudoannulata* was not significantly different between the control, linalool (1.0 μg/μL), and methyl salicylate (1.0 μg/μL), but the linalool (0.5 μg/μL) treatment was significantly greater than the control treatment (Figure 4).

### 3.3. Field Trapping

**Figure 5:**
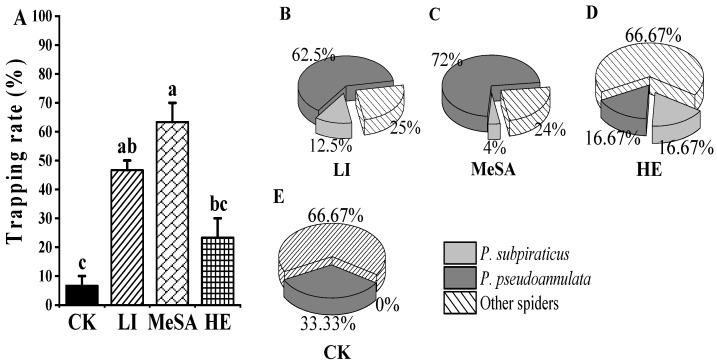
Field trapping rate and spider number: (**A**) = the trapping rate (traps with spiders/total traps), Columns starting with different lowercase letters are significantly different (ANOVA, Tukey *p* < 0.05), N = 3. (**B**–**E**) = the percentage of *P. subpiraticus*, *P. pseudoannulata*, and other spiders in the total number of spiders. CK = liquid paraffin, MeSA = methyl salicylate, HE = 2-heptanone, CH = cis-3-hexen-1-ol, LI = linalool.

The HIRVs were attractive to spiders, and the average catch of traps with HIRVs was higher than that of control traps. In particular, the traps containing methyl salicylate had higher catches than other traps. In the HIRVs traps, the proportions of *P. pseudoannulata* in the total spider number were methyl salicylate (72%), linalool (62.5%), and 2-heptanone (16.67%). The proportions of *P. subpiraticus* in the total spider number were 2-heptanone (16.67%), methyl salicylate (12.5%), and linalool (4%) (Figure 5).

## 4. Discussion

Spiders are among the most abundant predators in rice fields. *P. subpiraticus* and *P. pseudoannulata* are the predominant species of paddy field spiders. They have large populations and strong predatory ability [22,25]. HIPVs are specific volatile substances that plants release when they are damaged by pest feeding [38,39]. HIPVs are an important component of biological control and attract natural enemies to pest feeding locations [6,8]. The olfactometer is a useful tool that can simulate the emission of volatiles in a field environment and is useful for real-time observation of spider behavior [40]. Olfactometers have been widely used to determine the relationship between insect selection behavior and volatiles such as *Chilo suppressalis* Walker (Lepidoptera: Pyralidae) [41], *Cotesia urabae* Austin *et* Allen (Hymenoptera: Braconidae) [40], *Harmonia axyridis* Pallas (Coleoptera: Coccinellidae) [42], *Coccinella septempunctata* L. (Coleoptera: Coccinellidae) [43], *Diaeretiella rapae* McIntosh (Hymenoptera: Braconidae) [43], *Adoxophyes honmai* Yasuda (Lepidoptera: Tortricidae) [44], and *Diaphania indica* Saunders (Lepidoptera: Crambidae) [45]. We used a four-arm olfactometer to observe the selection behaviors of *P. subpiraticus* and *P. pseudoannulata* on four kinds of HIRVs. We obtained results for *P. subpiraticus* and *P. pseudoannulata* selection frequency and stay time using the four-arm olfactometer. These experiments have practical implications, because the selection behaviors of *P. subpiraticus* and *P. pseudoannulata* were strongly correlated with the types and concentrations of the attractants.

We found that 1.0 μg/μL methyl salicylate was significantly attractive to male *P. pseudoannulata*. Methyl salicylate is also attractive to the natural enemies of other pest insects. For example, Zhu and Park showed that methyl salicylate attracts *Coccinella septempunctata* L. (Coleoptera: Coccinellidae), a predator of *Aphis glycines* Matsumura (Homoptera: Aphididae) [46]. Boer et al. showed that methyl salicylate is attractive to the predatory mite *Phytoseiulus persimilis* Athias-Henriot (Acari: Phytoseiidae) [47]. James et al. demonstrated that methyl salicylate attracted *Chrysopa oculata* Say (Neuroptera: Chrysopidae) [48], *Chrysopa nigricornis* Burmeister (Neuroptera: Chrysopidae) [49], and Syrphidae spp. (Diptera: Syrphidae) [50]. Shimoda showed that methyl salicylate attracts the predatory mite *Neoseiulus californicus* McGregor (Acari: Phytoseidae), which is an important natural enemy of *Tetranychus urticae* Koch. (Acari: Tetranychidae) [51]. Our results showed that 1.0 μg/μL 2-heptanone was significantly attractive to male *P. subpiraticus.* Li et al. also found that 2-heptanone was strongly attractive to *Anagrus nilaparvatae* Pang *et* Wang (Hymenoptera: Mymaridae), the principal parasitoid of rice planthopper eggs [16]. Our results showed that linalool triggered spider olfactory responses. A 1.0 μg/μL linalool concentration was attractive to male *P. pseudoannulata*, while 0.5 μg/μL linalool was attractive to female *P. pseudoannulata* and male of *P. subpiraticus.* Linalool appears to be a very important attractant. Carroll et al. reported that *Spodoptera frugiperda* J. E. Smith (Lepidoptera: Noctuidae) was attracted to linalool [52]. Anderson et al. found that female *Bombyx mori* L. (Lepidoptera: Bombycidae) were attracted to linalool [53]. Reisenman et al. found that linalool could attract and stimulate oviposition in female *Manduca sexta* L. (Lepidoptera: Sphingidae) [54]. Cruz-Lopez et al. studied coffee volatiles and found that *Hypothenemus hampe* Ferrari (Coleoptera: Scolytidae) was attracted to linalool [55]. Mitra et al. found that female *Altica cyanea* Weber (Coleoptera: Chrysomelidae) were attracted to linalool [56].

Our research aimed to determine the influence of HIRVs on spider predation responses. Therefore, the attack latency and daily predation capacity were selected to evaluate the predation ability of *P. subpiraticus* and *P. pseudoannulata*. *Drosophila melanogaster* Meigen (Diptera: Drosophilidae) is a commonly used model organism in the laboratory. *D. melanogaster* is often used as a predator laboratory for alternative prey since it is easy to cultivate, rich in nutrients, and capable of flight [57,58,59,60]. Spiders generally only consume living prey and the lively *D. melanogaster* stimulate predation responses in spiders. In this study, *D. melanogaster* was used as an alternative prey for *P. subpiraticus* and *P. pseudoannulata*. The results showed that 1.0 μg/μL methyl salicylate significantly shortened the attack latency of male *P. pseudoannulata* and 0.5 μg/μL linalool significantly shortened the attack latency of *P. subpiraticus* and enhanced the daily predation capacity of female *P. pseudoannulata*. Lycosid spiders mainly live at the base of rice plants where high densities of brown planthoppers often occur. The brown planthopper is the target prey of many Lycosidae spiders [26]. After the brown planthoppers feed on rice, the rice releases methyl salicylate, linalool, and other volatiles [12,61]. These volatiles stimulate the olfactory selective response in spiders, shortening predation latency and increasing predation. This may be because spiders are attracted to the odor, which may affect their feeding response. Other studies on spider predation have shown that selected chemicals can shorten spider predation latency or enhance their predatory function. Suitable physiologically active plant substances can significantly enhance the predatory function of *P. pseudoannulata* on *N. lugens* [62,63]. Optimal low-dose pesticides, which shorten the subduing and feeding times of spiders on prey, will enhance insect control efficiency and the instant attack rate on the prey of *P. pseudoannulata*, *P. subpiraticus*, *P. astrigera* L. Koch (Araneae: Lycosidae), and *Coleosoma octomaculatum* Bösenberg *et* Strand (Araneae: Theridiidae) [64,65,66,67].

Spider density will affect the management of pest populations, and increasing the number of spider predators will aid in insect pest control. There are many examples of using pheromones to attract and retain natural enemies. Simpson et al. used methyl salicylate as an attractant for crops and found that parasitic wasps remain longer in these attractant crops [68]. Jaworski et al. also proved that placing methyl salicylate attractants in orchards increased the effect of predators in controlling pests compared to untreated orchards [69]. Our field experiments showed that the spider trapping rate, using methyl salicylate and linalool as attractants, was greater than that of the control treatment. Zhu and Park also found that traps baited with methyl salicylate were highly attractive to adult *C. septempunctata* in field tests [46]. These results show that natural enemies might use methyl salicylate as an olfactory signal to locate prey. The trapped *P. pseudoannulata* dominated, which showed that HIPVs not only attracts *P. pseudoannulata* in the laboratory but also attracts *P. pseudoannulata* in the field. The *P. subpiraticus* was trapped in smaller numbers, which may be because *P. subpiraticus* mainly wanders on the water surface and the base of rice plants bordering the water surface, while the traps used for this experiment were placed above the water surface. The other spiders trapped were mainly Salticidae and Clubionidae spiders, which are good at jumping, and they may have accidentally fallen into the trap. According to a preliminary field study, *P. subpiraticus* and *P. pseudoannulata* were the dominant species at the experimental site [70], but the number of trapped spiders was lower than expected. There are many uncontrollable factors that affect spider numbers, such as temperature, humidity, wind, the size and location of the trap, and the concentration of attractants.

In conclusion, our results indicate that HIRVs can attract spiders and increase predation. Two dominant species of rice spiders (*P. pseudoannulata* and *P. subpiraticus*) were attracted by one or two concentrations of methyl salicylate, linalool, or 2-heptanone. A predatory ability experiment showed that linalool and methyl salicylate shortened predation latency. Linalool increased the daily predation capacity of the spiders. A field experiment verified that methyl salicylate and linalool are attractive to spiders. In future research, we will determine the reasons why HIRVs enhance the predation ability of spiders and develop practical methods for using HIRVs to attract and retain spiders in rice fields.

## Figures and Tables

**Table 1:** Behavioral responses (observed frequencies (N = 30) compared to expected frequencies assuming random distribution by using a χ^2^ test) of spider to volatiles.

Spider	Sex	Treatment	χ^2^	*p*	Significance
*P. pseudoannulata*	male	CK	2.533	0.469	NS
		MeSA	8.667	0.034	*
		HE	9.200	0.027	*
		CH	6.533	0.088	NS
		LI	10.267	0.016	*
	female	CK	2.267	0.519	NS
		MeSA	1.200	0.753	NS
		HE	0.667	0.881	NS
		CH	3.867	0.276	NS
		LI	13.200	0.004	**
*P. subpiraticus*	male	CK	5.467	0.141	NS
		MeSA	3.867	0.276	NS
		HE	16.667	0.001	**
		CH	5.467	0.141	NS
		LI	13.467	0.004	**
	female	CK	7.600	0.055	NS
		MeSA	3.333	0.343	NS
		HE	4.400	0.221	NS
		CH	2.267	0.519	NS
		LI	2.533	0.469	NS

Note: CK indicates liquid paraffin, MeSA = methyl salicylate, HE = 2-heptanone, CH = cis-3-hexen-1-ol, LI = linalool; “NS” = no significant difference; “*” denotes a significant difference at the *p* < 0.05 level; “**” denotes a significant difference at the *p* < 0.01.

**Table 2:** Stay times of spiders in the four test areas.

Spider Name	Sex	Treatment	0.5 μg/μL	1.0 μg/μL	1.5 μg/μL	ck
*P. pseudoannulata*	male	CH	2.151 ± 0.457 a	1.323 ± 0.407 ab	0.661 ± 0.313 b	0.826 ± 0.343 ab
		MeSA	0.990 ± 0.368 b	2.313 ± 0.459 a	1.157 ± 0.381 b	0.494 ± 0.275 b
		HE	2.110 ± 0.450 a	0.534 ± 0.273 b	0.668 ± 0.312 b	1.652 ± 0.434 a b
		LI	1.171 ± 0.387 b	2.295 ± 0.456 a	0.822 ± 0.335 b	0.661 ± 0.313 b
	female	CH	1.986 ± 0.452 a	1.161 ± 0.391 a	0.993 ± 0.369 a	0.827 ± 0.343 a
		MeSA	1.157 ± 0.390 a	1.651 ± 0.434 a	0.992 ± 0.368 a	1.156 ± 0.389 a
		HE	1.161 ± 0.391 a	1.491 ± 0.423 a	0.990 ± 0.368 a	1.326 ± 0.409 a
		LI	2.640 ± 0.456 a	0.989 ± 0.367 b	0.656 ± 0.311 b	0.655 ± 0.310 b
*P. subpiraticus*	male	CH	0.827 ± 0.343 a	1.489 ± 0.424 a	0.661 ± 0.313 a	1.987 ± 0.452 a
		MeSA	1.156 ± 0.389 a	1.154 ± 0.390 a	1.821 ± 0.445 a	0.828 ± 0.344 a
		HE	0.825 ± 0.342 b	2.474 ± 0.460 a	0.661 ± 0.313 b	0.991 ± 0.368 b
		LI	2.647 ± 0.460 a	0.991 ± 0.368 b	0.496 ± 0.276 b	0.826 ± 0.343 b
	female	CH	0.993 ± 0.368 a	1.486 ± 0.421 a	0.836 ± 0.343 a	1.656 ± 0.434 a
		MeSA	1.159 ± 0.390 a	0.662 ± 0.313 a	1.324 ± 0.407 a	1.819 ± 0.443 a
		HE	1.167 ± 0.392 a	1.986 ± 0.451 a	0.662 ± 0.313 a	1.157 ± 0.389 a
		LI	1.820 ± 0.444 a	1.158 ± 0.389 a	0.828 ± 0.344 a	1.157 ± 0.386 a

**Note:** Data are mean ± SE; CK = liquid paraffin, MeSA = methyl salicylate, HE = 2-heptanone, CH = cis-3-hexen-1-ol, LI = linalool; different lowercase letters indicate significant differences in stay time in different areas (ANOVA, Tukey *p* < 0.05).

## Data Availability

Data are contained within the article.
